# Cell-Penetrating Peptides Derived from Animal Venoms and Toxins

**DOI:** 10.3390/toxins13020147

**Published:** 2021-02-15

**Authors:** Gandhi Rádis-Baptista

**Affiliations:** Laboratory of Biochemistry and Biotechnology, Institute for Marine Sciences, Federal University of Ceara, Fortaleza 60165-081, Brazil; gandhi.radis@ufc.br; Tel.: +55-85-996886054

**Keywords:** cell-penetrating peptide, venom peptide, arachnid venom peptide, insect venom peptide, snake venom peptide, cellular uptake, peptide chemical modification, peptide engineering, peptide design, peptide carrier

## Abstract

Cell-penetrating peptides (CPPs) comprise a class of short polypeptides that possess the ability to selectively interact with the cytoplasmic membrane of certain cell types, translocate across plasma membranes and accumulate in the cell cytoplasm, organelles (e.g., the nucleus and mitochondria) and other subcellular compartments. CPPs are either of natural origin or de novo designed and synthesized from segments and patches of larger proteins or designed by algorithms. With such intrinsic properties, along with membrane permeation, translocation and cellular uptake properties, CPPs can intracellularly convey diverse substances and nanomaterials, such as hydrophilic organic compounds and drugs, macromolecules (nucleic acids and proteins), nanoparticles (nanocrystals and polyplexes), metals and radionuclides, which can be covalently attached via CPP N- and C-terminals or through preparation of CPP complexes. A cumulative number of studies on animal toxins, primarily isolated from the venom of arthropods and snakes, have revealed the cell-penetrating activities of venom peptides and toxins, which can be harnessed for application in biomedicine and pharmaceutical biotechnology. In this review, I aimed to collate examples of peptides from animal venoms and toxic secretions that possess the ability to penetrate diverse types of cells. These venom CPPs have been chemically or structurally modified to enhance cell selectivity, bioavailability and a range of target applications. Herein, examples are listed and discussed, including cysteine-stabilized and linear, α-helical peptides, with cationic and amphipathic character, from the venom of insects (e.g., melittin, anoplin, mastoparans), arachnids (latarcin, lycosin, chlorotoxin, maurocalcine/imperatoxin homologs and wasabi receptor toxin), fish (pardaxins), amphibian (bombesin) and snakes (crotamine and cathelicidins).

## 1. Introduction

### Context

It has been established that the more diverse a biome is, the more intricate is the chemical-ecological relationships among organisms sharing the same niche and richer is the diversity of molecules and pharmacological activities that these co-inhabiting organisms contain. Indeed, a great probability of success in the drug discovery process from nature is connected to maximal biodiversity and chemical diversity [1].

Animal venoms and skin secretions are rich biological materials that contain a blend of biologically and pharmacologically active components, used by venomous and poisonous organisms for defense, predation and territorial disputes. The mechanisms via which these organisms deliver toxic components are related to how toxins have evolved and exerted their ecological functions [2]. As a result of millions of years of evolution, the diversity of molecular structures and activities is immeasurable, with hidden functions and structures only recently disclosed by modern omics techniques associated with classical pharmacological studies [3]. Thus, the holistic molecular genetic analyses of venomous animals utilizing, for example, transcriptome in combination with proteome studies of venom glands, have revealed not only the diversity of a set of substances produced by a given organism that inhabits a particular biome but also the uniqueness of such molecular repertoire expressed as active components of biological materials, such as venoms [4,5,6,7,8].

Although marine and terrestrial biological reservoirs of active venom peptides appear crucial sources for drug discovery, examples of basic and applied research should be provided to attract the attention of the scientific community both in academia and industry. Fundamental research, development and innovation of venom components as diagnostics, therapeutics or both are not always an obvious issue for consideration for the general audience. Furthermore, molecular diversity is intrinsically related to biodiversity and pharmaceutical success in drug discovery from nature is dependent on the abundance and variety of biological resources, thus highlighting arguments for policies of environmental conservation and economic sustainability worldwide [9].

A particular class of molecules from animal venom that has been under constant focus comprises biologically active (bioactive) peptides. Owing to their selectivity for corresponding cell receptors and target specificity, as well as the relative structural stability in body fluids and amenability to be genetically and synthetically engineered, natural venom peptides are promising scaffolds for conversion into biopharmaceuticals (biotherapeutics), as exemplified by cysteine-stabilized and linear helical peptides [10,11,12]. In this review, I discussed natural peptides from animal venom and their derivatives possessing intrinsic properties such as interaction with biomembranes of target cells, access to the cytoplasm through membrane translocation and eventual accumulation in distinct organelles such as the nucleus and mitochondria. This class of special peptides, collectively called cell-penetrating peptides (CPPs), comprise full sequences, peptide segments or patches of large proteins and even encrypted subcellular localization signals, as well as rationally designed peptide chimeras, cyclic, stapled, dimeric, multivalent and self-assembled peptides and peptoid foldamers [13,14,15].

In the field of toxicology, membrane translocation and cell-penetration capabilities are well-recognized phenomena mainly associated with microbial and plant toxins, such as binary bacterial toxins (e.g., diphtheria toxin, Shiga toxin and cholera toxin) and plant ribosome inhibitor proteins (e.g., ricin and abrin), which bind to their respective receptors on the eukaryotic cytoplasmic membrane and enter cells by receptor-mediated endocytosis [16,17,18]. Less common but with a steeply increasing number of reports published over the years, animal toxins and venom peptides endowed with intrinsic properties of membrane permeation and cell internalization are currently being unraveled. These venom CPPs and derivatives, recapitulated herein, mechanistically penetrate cells by distinct pathways that involve receptor-dependent and/or receptor-independent endocytosis (non-disruptive peptides) or direct translocation through pore formation, followed by cellular uptake and intracellular compartmentalization (disruptive peptides). Hence, in this review, I collated current examples of cell-penetrating peptides derived from animal venoms and toxins, their basic research and advanced applications.

Cell-penetrating peptides (CPPs) consist of short sequences that range from few amino acids to less than 40 residues, which owing to their physicochemical and biological properties can cross lipid membranes of cells and intracellularly transport diverse types of molecular cargoes in the form of covalent conjugates or noncovalent complexes. The first CPP sequences were characterized and derived from the transactivator of transcription from HIV-1 (Tat protein), the antennapedia (Antp) homeodomain from *Drosophila and* VP22 from Herpes simplex virus type I, almost three decades ago. For instance, CPPs such as Tat^48–60^ (residues 48–60, GRKKRRQRRRPPQ) and penetratin (Antp^43–58^, RQIKIWFQNRRMKWKK), which are short-derived fragments from TAT protein and *Drosophila* Antp, respectively, were able to translocate across the plasma membrane of eukaryotic cells and set the stage for the development of a new class of non-viral vectors for the intracellular delivery of compounds and, therefore, converted into archetypal CPPs [19,20]. Since these initial discoveries, the field of CPPs has expanded into a prolific field of research. Databases of curated information about experimentally characterized CPPs and algorithms to predict CPPs have been created, along with the expansion of the field of CPPs [21,22,23,24]. It has become evident that CPPs are mostly linear sequences but cyclic sequences have also been detected and developed. They are usually composed of positively charged amino acids with or without distributed hydrophobic residues, conferring these peptide structures with a variable but a considerable level of amphipathic and cationicity and high affinity for negatively charged lipid membranes and their components, including phospholipids and proteoglycans. In particular, in the case of linear CPPs, studies investigating the relationship between CPP secondary structures and penetrability have demonstrated that cationic helical peptides possess superior cell permeation than amphipathic helical and amphipathic random peptides, as revealed through synthetic polyarginine CPPs stabilized with different proportion of α-aminoisobutyric acid [25]. Nevertheless, unstructured peptides in solution eventually form helices upon interaction with plasma membranes, promoting their efficient membrane insertion, translocation and cellular uptake [26]. Hydrophobic interaction followed by membrane insertion also plays a role in the cell penetration property demonstrated by some amphipathic CPPs [27]. Importantly, from the perspective of applications in biomedicine and pharmaceutical biotechnology, CPPs can simultaneously mediate the intracellular delivery of diverse molecular cargos, along with the membrane-crossing activity. These molecular cargoes include hydrophilic drugs, radionuclides, imaging agents (fluorescent dyes), biopolymers (nucleic acids, polypeptides), functionalized liposomes and nanoparticles (nanocrystals, light-sensitive and magnetic nanoparticles). Therefore, CPPs have attracted considerable attention not only for their application as vectors for drug delivery but also as agents for diagnostics and therapy (theranostics) in medicine [28,29,30,31].

Regarding their mechanistic of cell penetrability, although not conclusively elucidated, it has been shown that CPPs traverse hydrophobic membranes and enter into cells through distinct routes, relying on both membrane-disruptive and non-disruptive processes, this later comprising energy-dependent, receptor-mediated and receptor-independent endocytosis, as well as energy independent, direct translocation strategies. Hence, pathways for CPP entry into cells involve: (I) non-disruptive endocytic routes, such as clathrin-mediated endocytosis, caveolae-mediated uptake, clathrin/caveolae-independent endocytosis and micropinocytosis; (II) non-disruptive membrane, direct translocation by alternative routes, like inverted micelle formation and “carpet” model penetration; (III) direct translocation by membrane-disruptive routes, such as pore-formation (toroid and barrel-stave pores) and electroporation-like permeabilization. Moreover, based on experimental evidence with numerous known CPPs, more than a single pathway of membrane permeation and cellular internalization may be involved, depending on the CPP physicochemical features, effective concentrations (i.e., the peptide-to-cell ratio) and targeted cell types [19,20,32]. Although distinct pathways are involved in membrane translocation and internalization of several distinct CPPs investigated, the mode of membrane interaction, the efficacy of CPP-mediated cargo-dependent transport, subcellular compartmentalization and eventual adverse cytotoxic effects, depend not only on their own CPP structures but also on the position and nature of the molecular cargoes and the types of cell targets, as observed with CPPs that were conjugated with fluorescent dyes and polypeptides [32,33,34,35,36].

Table 1 presents examples of CPP archetypes, their sequences, origins and physicochemical and structural attributes.

Interestingly, most structural and physicochemical features of CPPs can be observed in members of another class of bioactive peptides, termed antimicrobial peptides (AMPs), which are ubiquitously found in cells, tissues and biological fluids of organisms from diverse kingdoms, including venoms of poisonous animals [37]. Indeed, based on their structural characteristics, AMPs are membrane-active peptides that possess the ability to disrupt the plasma membrane integrity of their respective cellular targets as their principal mechanism of action, and penetrate target cells through pores [38,39]. The cell penetrability of AMPs appears to reinforce their antimicrobial efficacy owing to the interaction and interference with intracellular components, including macromolecules and organelles [40]. In this case, cellular uptake and permeability of AMPs can be associated with variable levels of cytotoxicity. However, the design and synthesis of AMPs can provide engineered peptides with tuned membrane translocation and cellular uptake capabilities, combined with lower or absent detrimental effects on membrane stability and cell viability, as exemplified by buforin II from the stomach tissue of the Asian toad *Bufo bufo garagrizans* and derivates [41] and other AMPs [42], including some presented in this article, in following sections.

To date, native venom peptides with cell-penetrating properties have been identified in a limited number of species of insects (bees and wasps), arachnids (spider and scorpions), fishes, amphibians and snakes (elapids and pit vipers). These CPPs have dual or multiple biological activities and are derived from the venoms or skin secretions, which are toxigenic, cytotoxic or antimicrobials. Thus, in this review, the origins, basic research and advances regarding these venom peptides and related sequences, as well as their original single, dual or multiple biological activities, including cell-penetrating properties, are presented and discussed. These venom CPPs include melittin from the honeybee, anoplin and mastoparans from wasps, latarcin and lycosin from spiders, chlorotoxin, maurocalcine and imperatoxin from scorpions, cardiotoxin, crotamine, crotalicidin and elapid cathelicidin-related antimicrobial peptides (CRAMPs) from snakes, pardaxin from fish skin and bombesin from frog skin, all presented in following sections. The importance of this theme and class of bioactive peptides relies on the fact that venom peptides and toxins, as well as related sequences, have evolved for millions of years of natural history and can be considered crucial improvements in peptide design that efficiently performs their functions. Thus, recombinant products, peptide chimeras and synthetic analogs can be engineered and prepared for biomedical applications, as well as potentially converted into biopharmaceuticals.

## 2. Cell-Penetrating Peptides from Animal Venoms and Toxins

An increasing number of studies have reported on the applicability of venom peptides in the diagnosis and prospective treatment of diseased processes that are dependent on selective and specific cell membrane interaction and/or receptor binding, translocation across the cell membrane (cell penetration), intracellular trafficking and subcellular localization to exert their effect. For instance, the antitumor activity of scorpion neurotoxins, which are venom peptides that particularly act on ion channels, has been established [43]. Furthermore, venom peptides that possess cell-penetrability properties and druggability have been identified from the venom of other arachnids and insects. The first two subsections outline well-documented examples of venom peptides from honeybees, wasps, spiders and scorpion with cell penetration features. The following subsections discuss venom peptides with cell penetration ability derived from fish, amphibians and snakes. These examples of CPPs characterized from animal venoms and toxins will illustrate their multifaceted biological activities and their potential and practical application in clinics and biomedicine. Table 2, Table 3 and Table 4 summarize venom peptides and derivatives with cell-penetrating properties, their overall structures and mode of cell entry, as discussed in the text.

### 2.1. CPPs from Insects

#### 2.1.1. Melittin

Melittin is the major peptide component of the European honeybee Apis mellifera venom, comprising up to 60% of the dried crude venom. This cationic peptide, with a primary sequence consisting of 26 amino acid residues (GIGAVLKVLTTGLPALISWIKRKRQQ-NH2) segmented in a hydrophobic C-terminus followed by a hydrophilic N-terminus, adopts an amphipathic α-helix topology that is typical of membrane-interacting and lytic peptides [44,45]. Owing to its structural conformation, melittin, at low concentrations, inserts into neutrally charged lipid bilayers and phospholipid membranes, such as the AMP indolicin-derivatives and the Arg-rich TaT CPP. After membrane insertion, melittin forms transmembrane pores at higher concentrations, as observed by direct visualization using in situ atomic force microscopy and dynamic computer simulation [46,47]. The direct physical and chemical membrane-disrupting and cytolytic effects endow melittin with broad-spectrum anti-infective and anticancer activities, thus presenting the concept of converting cytolytic venom-peptides into safe therapeutics [44,45,48]. Reportedly, fluorescent dye (AlexaFluor 430)-conjugated peptides with both the monomeric and dimeric forms of melittin can penetrate human gastric cancer (NUGC-3) cells and accumulate in the nuclear membrane, causing shrinkage of the nucleus and contributing to the cell death effect [49]. In addition to a variety of pharmaceutical formulations and derivatization, which have been conceived to improve the druggability of melittin and avoid side effects of hemolysis and nonspecific damage to healthy cells [48,50,51], melittin derivatives and short peptide analogs have also been designed with disruptive and non-disruptive cell membrane penetrability. For instance, a chimeric peptide was developed that fused the Tat11 CPP and CM18 hybrid (KWKLFKKIGAVLKVLTTG, residues 1–7 of cecropin-A and 2–12 of melittin), which combined plasma membrane translocation and endosomal escape and was successfully applied for the delivery of several types of cell-impermeant cargoes [52]. The cell-penetrating activity of the chimeric melittin peptide constructs has been investigated in terms of the directional target and selective death of tumor-associated M2-type macrophages. Hence, a chimeric peptide hybrid consisting of melittin fused via a GGGGS linker to the pro-apoptotic peptide d(KLAKLAK)_2_ (i.e., GIGAVLKVLTTGLPALISWIKRKRQQGGGGS-d[KLAKLAKKLAKLAK]) efficiently induced the selective mitochondrial-mediated apoptotic cell death of M2 macrophages in vitro and (CD206^+^) M2-like tumor-associated macrophages in vivo, reducing tumor growth rates, tumor mass and angiogenesis [53]. In another interesting example, melittin-derived peptides were developed, with C-terminal sequences modified to include Arg and His residues for peptide-nucleotide interaction and pH sensing, resulting in the peptides p5RHH (VLTTGLPALISWIRRRHRRHC) and p5RWR (VLTTGLPALISWIKRKRQQRWRRRR). Notably, p5RHH arrested proliferation of immortalized and cancer cells, to inhibit metastasis in a cancer mouse xenograft model, as well as transfected cells and delivered siRNAs into the cytoplasm [54,55]. Truncated versions of melittin were reported to enhance the pH-dependent endosomal escape of DNA-polymer vector complexes (polyplexes) and, therefore, the transfection efficiency [56]. As demonstrated in this study, the truncated melittin derivatives, consisting of residues 1 to 20 (MT20, GIGAVLKVLTTGLPALISWI and FL20, GIGAILKVLATGLPTLISWI) showed low hemolytic activity at neutral pH but were membranotlytic in the acidic milieu of endosomes. These melittin-derived peptides did not interfere with the formation and stability of branched polyethyleneimine DNA polyplexes and improved transfection and DNA delivery in immortalized and cancer cells. Moreover, melittin fragments have been shown to enhance the cellular uptake of macromolecules. In several eukaryotic cell lines, the N-terminal melittin fragment 1 to 14 (GIGAVLKVLTTGLP) demonstrated high efficiency in the internalization of fluorescent nanocrystals and large proteins [57].

#### 2.1.2. Anoplin

Anoplin (GLLKRIKTLL-amide) is a short (10-residue), amphipathic, α-helical antimicrobial peptide purified from the venom of the Japanese solitary wasp Anoplius samariensis [58]. The membrane interaction of anoplin was verified using lipid monolayers imaged using atomic force microscopy and X-ray spectromicroscopy [59]. Owing to its simple structure and a broad spectrum of biological and pharmacological activity, anoplin has attracted research interest in terms of chemical manipulation and structural modification, to modulate its activity and bioavailability, as recently reviewed [60]. Particularly, the C-N terminal dimerization of anoplin not only showed superior stability and performance when compared with the parent peptide but also and importantly, enhanced inner membrane permeability and DNA binding ability [61]. These improved properties and performance of structurally modified anoplin have inspired the design of self-assembling anoplin derivatives for selective and sustained antibiosis [62], as well as conceived in situ tumor microenvironment-induced self-assembly of polymer–anoplin conjugates to attack solid tumors and analogous with other peptide-functionalized nanoconjugates [60,63].

#### 2.1.3. Mastoparan

Mastoparan (INLKALAALAKKIL-amide) is a cationic tetradecapeptide with an amphipathic character derived from the venom of the Korean leather-jacket Vespula lewisii that corresponds to up to 50% of the crude venom and has demonstrated mast cell degranulation when injected [64]. A highly similar peptide (mastoparan-X, INWKGIAAMAKKLL-amide) was subsequently characterized from the venom of the Japanese hornet V. xanthoptera [65]. Mastoparan and related-peptides have served as a versatile structural scaffold to design diverse CPPs, as exemplified by the mitoparan-serie and transportans [66,67,68]. Transportan (GWTLNSAGYLLGKINLKALAALAKKIL-amide) is a designed, 27-residue chimeric peptide that was constructed by joining the N-terminal residues 1 to 12 of the neuropeptide galanin (GWTLNSAGYLLG) via a lysine residue (Lys^13^) to the mastoparan 14-residue sequence [66]. Transportan 10 (AGYLLGKINLKALAALAKKIL-amide) is a deletion analog of transportan [67]. In aqueous solution, both mastoparan and transportan present a disordered structure, which is converted into a α-helical structure upon interaction with micelles and binding to lipid membranes [69]. The mechanism of membrane interaction, binding and cell penetration is essentially the same for mastoparans and transportan, as quantitatively demonstrated with biomembrane models [70]. Transportan and transportan 10 are two of the most studied designed mastoparan-derived peptides that possess significant cell-penetrating properties. The 27-residue transportan, galanin-mastoparan chimera, was biotinylated at Lys^13^ and radiolabeled with I^135^ to assess its cellular uptake, intracellular distribution and localization. As detected with biotinyl-transportan and ^125^I-biotinyl-transportan, the cell penetration of this peptide is highly efficient, energy- independent and occurs rapidly (<30 min). Transportan traverses the plasma membrane and is also endocytosed by several lines of eukaryotic cells, entering the cytoplasm and accumulating in the nucleus and subnuclear structures. Moreover, Lys^13^ serves as a connector for attachment molecular cargoes by covalent conjugation [66]. It was shown that increasing the charge and amphipathicity of transportan 10 but preserving the helical structure of the mastoparan stretch, like in transportan 10–5 (AGYLLGKINLKKLAKL(Aib)KKIL-amide), resulted in analogs with improved penetrability and preference for cancer cells over healthy cells [71]. Transportan and transportan-derived CPPs have been investigated for various prospective biomedical applications. For instance, PepFecs, stearyl-derivatives of TP10, are low cytotoxic and immunogenic CPPs endowed with endosomal escape properties and the ability to deliver nucleic acids both in vitro and in vivo; hence, these analogs not only improved the efficiency of transport and delivery of therapeutic agents but also decreased the risks of adverse effects [72]. A chimeric transportan, namely Transportan 9dR (GWTLNSAGYLLGKINLKALAALAKKILdRdRdRdRdRdRdRdRdR, where dR = D-arginine), was devised and used to form a complex and deliver siRNAs that target a gene segment of influenza virus H1N1 into epithelial cells of the respiratory tract, with efficient inhibition of viral replication achieved both in vitro and in vivo [73]. Microemulsions containing paclitaxel loaded with transportan showed improved penetrability and enhanced delivery, with superior efficacy of the antitumor drug against cutaneous tumor cells in culture and in skin cancer models [74]. In addition to these examples of cellular uptake and cargo delivery, transportan 10 and its analogs display significant antimicrobial activity against multi-drug resistant and clinically relevant bacteria, mediating their effects by means of membrane perturbation, ligation to lipopolysaccharides and binding to intracellular DNA [75,76]. Notably, vancomycin covalently linked to transportan 10 at is C-terminal, as in the conjugates AGYLLGK7(C(O)-Tra(1,4)-PEG4-C(O)-Van)INLKALAALAKKIL-amide and AGYLLGKINLKALAALAKKIL-Ala(Tra(1,4)-PEG4-C(O)-Van)-amide (where PEG4 is the linker group and Van is vancomycin), substantially increased the antibacterial activity of the conjugates against intracellular or extracellular compartmentalized multi-drug resistant bacteria in vitro. Moreover, transportan-vancomycin conjugates, including [Lys7(PEG4-Van)]TP10 and its fluorescent analog, translocated across the blood-brain barrier and accumulated in the cerebral tissue in an in vivo mouse model [77].

Mitoparan (INLKKLAKLAbiKKIL-amide), the [Lys^5,8^, Aib^10^]mastoparan, was essentially designed by substituting Ala residues 5, 8 and 10 of mastoparan with Lys^5,8^ and Aib^10^ (α-aminoisobutyric acid), resulting in analogs with lower cytotoxicity and better pharmacodynamic and intracellular target selectivity [68]. Mitoparan is a more potent secretagogue and cytotoxic agent than mastoparan, able to efficiently translocate across the plasma membrane of mammalian cells and localize to the mitochondria, where it can trigger apoptosis-mediated cell death [78]. Enantiomer analogs of mastoparan and mitoparan, such as the inverso (all D-amino acid residues), retro (reverse sequence) and retro-inverso, were synthesized to investigate the relationship between structure and activity, that is, cellular penetrability, mast cell degranulation ability and cytotoxicity [79]. Interestingly, all D-isomers of mastoparan and mitoparan preserved their intrinsic biological activities, while all D-mastoparan showed better translocation efficacy (cell penetrability), exceeding that of mitoparan, with reduced cytotoxicity and resistance to proteases. Reportedly, mastoparan and mitoparan cause mitochondrial depolarization and activation of the caspase cascade and kill cancer cells. Furthermore, an analog of mitoparan [Z-Gly-RGD(DPhe)-mitP] that consists of Z-glycine, the integrin-specific motif RGD and a D isomer of phenylalanine was developed to treat psoriasis, as well as to compartmentalize into the mitochondria and modulate its activity in transformed aneuploid immortal keratinocyte (HaCaT) cells from adult human skin [80].

### 2.2. CPPs from Arachnids

#### 2.2.1. Latarcin-1

The latarcins comprise a group of membrane-interacting peptides from spider venom that possess cytolytic and antimicrobial properties, with potential cell-penetrating activity. Latarcins are 25- to 30-residue peptides, rich in Lys and Arg, derived from the venom of the spider Lachesana tarabaevi [81]. Short latarcin 1-derived decapeptide (KWRRKLKKLR) was designed using an algorithm, labeled using fluorescein isothiocyanate (FITC) and shown to be cytotoxic to human cervix cancer (HeLa) cells, with low levels of cellular uptake. However, prolongation of its C-terminus by chemical conjugation with the nuclear localization sequence (NLS) from Simian Virus T40 antigen (PKKKRKV) resulted in a chimeric latarcin 1-derived peptide (KWRRKLKKLRPKKKRKV), which displayed improved cell translocation efficiency and lower cytotoxicity, as observed by the accumulation of the fluorescent analog in lysosomes after endocytosis. Moreover, the chimeric latarcin-derived peptide was efficiently transported and delivered into the cytoplasm of adherent cells, along with a large reporter enzyme (β-galactosidase) in its active form [82]. In this case, although cell-penetration was enhanced by peptide engineering, i.e., by conjugation of an NLS, the intrinsic biological activity of the latarcin-derived peptide appeared to be essential for use as bivalent AMP/CPP. Indeed, this dual activity of latarcin 1-derived peptides was demonstrated with FITC-labeled analogs in spores and hyphae of the plant pathogenic fungus Fusarium solani, in which the NLS conjugated at the peptide C-terminal contributed to enhancing the internalization of the chimeric peptide, with selective and variable level of antifungal activity [83].

#### 2.2.2. Lycosin-I

Lycosin-I is a linear, cationic, 24-residue peptide (RKGWFKAMKSIAKFIAKEKLKEHL) from the venom of the spider Lycosa singorensis that forms an amphiphilic α-helix upon interaction with lipid membranes. Several studies have demonstrated the antibacterial and antitumor activities of lycosin-I, along with its ability to selectively enter into the cytoplasm of tumor cells and activate antiproliferative and cell death signaling [84,85,86]. Tan and colleagues [87] quantitatively demonstrated, by single-molecule fluorescence imaging with internal reflection fluorescence microscopy, the time-dependent aggregation and diffusion of rhodamine B-labeled lycosin-I on a neutral lipid membrane model as part of its mechanism of cell penetration, as well as anticancer activity and selectivity. Owing to the cell penetration and antitumor activities, lycosin-I was used to functionalize gold nanoparticles, to target and penetrate tumors, as confirmed in vitro and in vivo with different tumor cell lines and a mouse xenograft tumor model, respectively. Moreover, as this spider venom peptide preferentially accumulated in tumor cells and tissues, lycosin-I functionalized gold nanoparticles were efficiently used to kill cancer cells by the photothermal conversion effect under near-infrared irradiation [88]. Notably, the substitution of all Lys residues in lycosin-I peptide with Arg resulted in the R-lycosin-I (Ac-RGWFRAMRSIARFIARERLREHL-amide), which displayed improved anticancer activity and cellular penetrability against solid tumor cells, as assessed by employing unlabeled peptides and fluorescent-labeled analogs, prepared by conjugation with cyanine 5 (Cy5-lycosin-I) and FITC (FITC-R-lycosin-I) [89].

#### 2.2.3. Chlorotoxin

Chlorotoxin (“CTX,” MCMPCFTTDHQMARKCDDCCGGKGRGKCYGPQCLCR) is a 36-residue neurotoxin with the typical inhibitor cystine knott (ICK)/knottin fold, derived from the venom of the Israeli yellow scorpion Leiurus quinquestriatus [90]. CTX is one of the few known examples of neurotoxic peptides that exert its effect on small-conductance epithelial chloride ion channels but it also binds with high affinity to the matrix metalloproteinase 2 (MMP2) receptor on the target glioma cell membrane in neuroblastoma, avoiding healthy glial cells [91]. Owing to the high specificity and selectivity for glioma cells that overexpress MMP2, the synthetic version of CTX (designated, TM-601) has been used in clinical imaging and therapy of malignant glioma tumors, as exemplified by I^131^-iodination of the Tyr residue of the peptide, which produces I^131^-TM-601 used in radiodiagnostics and radiotherapy [92]. The multimodality of CTX in terms of its application in diagnosis and prospective therapy, as bioconjugates and theranostic agents to image and treat glioma was recently reviewed [93]. Accordingly, bioconjugates of CTXs include, for instance, crystal and magnetic nanoparticles, polymer-blend dots, in addition to the radiolabeled I^131^-TM-601. Notably, the compact and tight structure endows CTX with the ability to cross the blood-brain barrier and accumulate in glioma tumors. The routes of entry, intracellular trafficking and subcellular localization of CTX (TM-601) in glioma cells was compared with healthy cells using unlabeled and fluorescently labeled (Alexa Fluor 488) TM-601 [94]. Accordingly, cellular uptake of TM601 in U373 glioma cells was a rapid concentration- and time-dependent process, which was affected by chlorpromazine, a pharmacological inhibitor of clathrin-mediated endocytosis, involved in the transport of coated pits. Interestingly, as in the case of lycosin-I from spider venom, the replacement of Lys with Arg in the CTX sequence, as observed with the synthetic analogs [K15R/K23R]CTX and [K15R/K23R/Y29W], enhanced its cellular uptake, as demonstrated in human cervical carcinoma (HeLa) cells with fluorescent derivatives [95].

#### 2.2.4. Maurocalcine

Maurocalcine (“MCa,” GDCLPHLKLCKENKDCCSKKCKRRGTNIEKRCR) is a 33-amino acid long, cationic peptide isolated from the venom of the Tunisian scorpion Scorpio maurus palmatus. In solution, MCa folds to adopt the canonical ICK/knottin motif, in a three-stand arrangement (βββ) constrained by three disulfide bonds. The main biological activity of MCa is to activate the ryanodine receptor (RyR) to induce, within seconds, Ca^2+^release from the sarcoplasmic reticulum (SR) of myotubes in the skeletal muscle [96,97]. To exert its pharmacological action on the RyR channel and release calcium ions from intracellular stores, MCa translocates across the plasma membrane and accumulates in the cytoplasm and the nucleus of cells [98]. Furthermore, it was observed that the fast kinetic cellular uptake of non-covalently linked MCa complexes with streptavidin–cyanine 3 (Cy3) or cyanine 5 (Cy5) is independent of metabolic energy or endocytosis but needs to interact with negatively charged lipids (e.g., gangliosides and/or phosphatidylserine) on membrane surface domains for translocation, as well as with the negative potential of cells [99]. Similar to some CPPs, in addition to transporting large complexed cargoes such as streptavidin, MCa delivered other kinds of molecules inside cells, including the chemotherapeutic drug doxorubicin into human breast cancer cells [100,101]. This disulfide-rich peptide with relatively large size, at first glance, presents apparent disadvantages, including the need for conversion into carrier vectors for delivery of cell-impermeant compounds and nanoparticles; accordingly, disulfide-less and short derivatives of MCa, including a non-toxic all D-amino acid isomer, were prepared and investigated [102,103,104]. For instance, all D-isomers and disulfide-less MCa lacked pharmacological activity but preserved cell penetrability, while unstructured short analogs revealed variable but efficient levels of cellular penetrability and uptake [103,105]. Despite these shortcomings of structural downsizing and necessity to circumvent the in vitro folding of synthetic full-size MCa, the disulfide bond-cross-linked ^125^I-Tyr-MCa (an MCa analog with the three native disulfide bridges but an extra N-terminal Tyr residue for I^125^-radiolabeling) was still the most stable version detected in the bloodstream of intravenously injected mice when compared with the linear version (Lin-^125^I-Tyr-MCa) that degrades rapidly. Moreover, after biodistribution, the ^125^I-Tyr-MCa concentrated in diverse tissues, mainly in the thyroid, except for the brain, owing to its inability to cross the blood-brain barrier [106]. Maurocalcine covalently conjugated with platinum and gold nanoparticles and functionalized with MCa-derived peptides have been prepared for biopharmaceutical applications [107,108].

#### 2.2.5. Imperatoxin

Imperatoxin A (“IpTxa,” GDCLPHLKRCKADNDCCGKKCKRRGTNAEKRCR) is a 33-residue in length, cationic, ICK-like peptide from the venom of the African scorpion Pandinus imperator, which reversibly activates the RyR to release calcium ions from the SR into the cytoplasm of muscle cells. Owing to the sequence identity and structural similarity with maurocalcin, Gurrola and colleagues prepared fluorescent derivatives of IpTxa, with Alexa Fluor 546 covalently conjugated to the thiolated peptide and confirmed that this scorpion venom toxin traversed the cell membrane and internalized into mouse ventricular cardiomyocytes in vitro to exert its intracellular effect on the RYR [109].

#### 2.2.6. Hadrucalcin

Hadrucalcin (“HdCa,” SEKDCIKHLQRCRENKDCCSKKCSRRGTNPEKRCR), a basic 35-residue peptide purified from the venom of the Mexican scorpion Hadrurus gertschi, like maurocalcine and imperatoxin, is an activator of RYRs [110]. Like its congeners, HdCa traversed the plasma membrane of cardiac ventricular myocytes and activated the skeletal RYR (RYR1) of the SR, rapidly releasing intracellular calcium for muscle contraction. Although speculative, the authors who first characterized HdCa [110] believed that the pentapeptide sequence KKCXR, present in all members of the calcin family, might be the segment responsible for cell penetration of this peptide, despite the uniform distribution of positive residues in the peptide structure and overall low amphiphaticity of HdCa. Nevertheless, from the HdCa, a short size cell-penetrating peptide (“HadUF_1−11_, H11”, SEKDAbu_5_IKHLQR_11_-C) was designed and used to functionalize quantum dot (QD) nanoparticles in combination with PEG5-CaRuby [111]. This biofunctionalized QD-CaRuby-H11 efficiently penetrated Baby hamster kidney (BHK21) cells in culture and served as a nanobiosensor to detect transient fluctuations in intracellular calcium levels in BHK21 and HEK293 cells expressing Ca^2+^-permeable N-methyl-D-aspartate receptors (NMDARs), by employing through fluorescence resonance energy transfer (FRET) measurements and total internal reflection fluorescence (TIRF) imaging.

#### 2.2.7. Wasabi Receptor Toxin

From the venom of the Australian Black Rock scorpion Urodacus manicatus, Lin King et al. [112] characterized a unique venom peptide (Wasabi receptor toxin, WaTx), with a mechanism of action that resembles that of plant irritants (piquants from allium and mustard plants) and acts via the chemosensitive TRPA1 ion channel present in afferent sensory neurons. Using a combination of electrophysiology, intracellular calcium imaging and FRET, with Alexa Fluor 488 labeled-WaTx and AlexaFluor568-IgG-loaded liposomes, it was observed that WaTx modulated intracellularly the TRPA1 channel activity after translocation across the lipid membrane. WaTx (ASPQQAKYCYEQCNVNKVPFDQCYQMCSPLERS), with 33 residues, is a κ-KTx scorpion toxin-like peptide that adopts a stable helical hairpin fold maintained by two disulfide bonds (C1-C4–and C2-C3) and the basic patch at the open end of the cysteine-stabilized helical hairpin was postulated as the cell-penetrating motif of WaTx.

Table 3 summarizes the peptides and derivatives from arachnid (spider and scorpion) venoms with cell-penetrating and cargo delivery capacity.

### 2.3. CPPs from Fish

#### Pardaxins

Pardaxins (P1 to P5) comprise a group of potent pore-forming, ichthyotoxic peptides that are secreted into seawater by the Mose sole fishes of the genus Pardachirus (P. marmoratus and P. pavoninus), all possessing similar structures composed of a single-chain acidic peptide of 33-residue, with an N-terminal hydrophobic α-helix connected via a dipeptide (SerPro) to a C-terminal amphiphilic α-helix (helix-hinge-helix) [113,114]. Natural or synthetic peptides are inserted into phospholipid-containing membranes, forming pores and inducing the cytolysis of both healthy and tumor cells, as well as the death of Gram-negative and Gram-positive bacteria [115,116]. Amino acid substitution, as seen in the second helix of pardaxins P4 and P5 and acylation of N-terminal Gly of P5, reportedly modulate membrane insertion and cytotoxicity [116,117]. In this respect, the conjugated cyanine 3 acylated pardaxin (Cy3-pardaxin 5) loses its cytotoxicity but retains its ability for membrane insertion, importantly, resulting in internalization and accumulation in the nucleus of neuroblastoma (NG108-15) and bovine chromaffin cells [117]. 

### 2.4. CPPs from Amphibian

#### Bombesin

Bombesin (EQRLGNQWAVGHLM-NH_2_) is a neuroactive tetradecapeptide originally isolated from the skin of the European fire-bellied toad Bombina bombina by Erspamer and collaborators (1971) [118]. Bombesin is included in this review as, like venoms, frog skin secretions usually contain defensive neuroactive and cytolytic peptides (e.g., opioid peptides, triptopyllins and AMPs) that act in concert to intoxicate the endocrine and nervous system of predators; hence, they are potentially toxic to vertebrates and mammalian organisms [119]. Bombesin adopts an environmental-dependent topology that supports a hairpin turn at the N-terminus and helical structures at the C-terminus [120]. Reportedly, bombesin binds with high affinity to the gastrin-releasing peptide receptor (GRPR) of target cells and the short C-terminal fragments (residues 7 to 14, WAVGHLMH-amide), sharing identity with gastrin-releasing peptide (GRP), was found to be crucial and sufficient for receptor binding, as well as the biological activity of bombesin-like peptides [118]. The bombesin/GRP receptor subtype 2 is known to be overexpressed in diverse cancer types, allowing the application of radiolabeled bombesin-like peptide analogs as tracers for the detection and/or treatment of G-protein-coupled receptor (GPCR)-positive tumors [121,122]. For instance, short bombesin-like peptide analogs with natural and non-natural amino acids have been synthesized and labeled with radionuclides (99 mTc, 111In, 90Y, 64Cu, 177Lu, 68Ga or 18F) and evaluated in vivo and in vitro in animal models and humans to detect GPCR-overexpressing tumors [121,123]. A formulation of multivalent Tat^49–57^-Lys3-bombesin^1-14^ chimera-functionalized 99mTc/177Lu-labeled gold nanoparticles (99mTc/177Lu-AuNP-Tat-BN) was prepared and internalized efficiently into the nucleus of prostate cancer (PC3) cells, relocating to the membrane after uptake of cytoplasmic accumulated bombesin, allowing the application of plasmonic photothermal therapy and targeted radiotherapy in prostate tumors [124].

### 2.5. CPPs from Snakes

#### 2.5.1. Crotamine

Crotamine, the first snake venom peptide known to selectively penetrate eukaryotic cells, is a 42-residue cationic peptide purified from the venom of certain populations of the South American rattlesnake Crotalus durissus terrificus and other subspecies of C. durissus [125,126,127]. Pharmacologically, at the molecular level, crotamine is essentially a neurotoxin that exerts its effect at nanomolar concentrations on voltage-dependent Na^+^ and K^+^ ion channels of different types of muscle cells [128,129]. At higher toxic concentrations, it causes myonecrosis owing to a massive influx of sodium ions into the skeletal muscle cells of injected mice and is referred to as small basic myotoxin [130]. Structurally, crotamine (YKQCHKKGGHCFPKEKICLPPSSDFGKMDCRWRWKCCKKGSG) is constrained by three disulfide bridges that result in an αβββ-fold, highly similar to that adopted by the human antimicrobial peptide β-defensin 2 and a restricted number of known toxins, despite differences in their primary sequences [131]. The β-defensin fold is a structural variant that is derived from a core motif generally present in peptides that are membrane-interacting and host defense effectors [132]. The high content of basic amino acid residues (nine lysines and two arginines) imparts crotamine a net positive charge and high cationicity, at physiological pH, demonstrating important physicochemical features of its membrane interaction and translocation. Indeed, the membrane-modifying properties of crotamine were studied with monolayer and planar lipid bilayer of phospholipids, as well as with ex vivo basement membranes of the chicken retina, thus confirming crotamine oligomerization, membrane insertion, pore formation and lipid membrane-fusogenic properties [133,134]. 

The cell penetration property of crotamine, in addition to its antiproliferative activity and other functionalities, has been reviewed in several articles recently published [135], as well as in the last decade [136,137,138]. The penetrability of crotamine was observed with fluorescent cyanine 3-labeled native peptide and diverse cell types in culture, including mouse blastocysts and embryonic stem cells [139]. Cyanine 3-conjugated crotamine, at low micromolar non-toxic concentrations, selectively and rapidly penetrated (within minutes) actively proliferating cells, particularly those between the S/G2 and G2/M phases of the cell cycle; intracellularly, this conjugate bound to subnuclear components such as centrioles and chromosomes. The selective cellular uptake of crotamine was shown to depend on the endocytic route after its initial interaction with negatively charged heparan sulfate proteoglycans on the plasma membrane of target cells [140]. Moreover, crotamine binds to nucleic acids and forms stable peptide-DNA complexes [140,141] intracellularly and in culture; accordingly, the intrinsic property of crotamine involves the unique functionality of a peptide vector to induce gene transduction of dividing cells. Reportedly, crotamine selectively interacts with and internalizes actively dividing proliferative cells, including cancer cells of epithelial origin in culture such as murine melanoma (B16-F10), human melanoma (SK-Mel-28) and human pancreatic carcinoma (Mia PaCa-2) cells, and its penetrability, tumor homing after biodistribution and anticancer activity, demonstrated in vivo using a murine model with engrafted subcutaneous melanoma [142]. The cell-penetrating efficacy of crotamine, associated with its anticancer activity, has been revealed in several in vitro and in vivo studies, with the subcellular and molecular details of its mode of action ascertained [143,144].

Downsizing of crotamine produced short peptides such as CyLoP derivatives and nucleolar targeting peptides (NrTPs), with variable efficacy allowing translocation into the plasma membrane and localization to different cell compartments. CyLoPs (cytosol-localizing peptides) such as CyLoP-1 (CRWRWKCCKK) were designed based on the two encrypted nuclear localization signals predicted from the crotamine primary sequence: residues 2 to 28 (KQCHKKGGHCFPKEKIC) and 27 to 39 (KMDCRWRWKCCKK) [145]. CyLoPs explicitly accumulate in the cytosol, endosome and nuclear perimeter of distinct cells investigated. In contrast, the NrTPs mimic most crotamine features, such as rapid cellular uptake, homing to the nucleus and binding to mitotic chromosomes, while improving other features, such as a reduction in the peptide size and accumulation in the nucleolus. For instance, NrTP-1 (YKQCHKKGGKKGSG), a prototype of NrTP-serie, was designed based on the secondary structure of crotamine instead of the primary sequence, by splicing the spatially close Gly^9^ and Lys^38^. The flexible Ahx connector was included in one of the analogs, namely, NrTP-2 (YKQCHKKGG-Ahx-KKGSG), which was later proved to be dispensable for cellular penetrability of this crotamine derivative [146]. As demonstrated by specific pharmacological inhibitors of cell internalization pathways, NrTP-1 selectively and efficiently penetrated human pancreatic (BxPC-3) and human ductal mammary gland (BT-474) carcinoma cells, predominantly via clathrin-dependent endocytosis, although other entry portals were also employed [147]. The NrTP-1 prototype was further modified to produce analogs and investigate their structure-activity relationships. Overall, in a series of sequential studies, replacing the cysteine residue at position 4 in NrTP-1 for a serine resulted in an analog, namely NrTP-6 (YKQSHKKGGKKGSG), with a significantly improved performance in terms of its interaction with biomembranes, penetrability, cargo transportation of large macromolecules, concentration-independent internalization kinetics and negligible cytotoxicity to healthy cells [148,149,150,151]. The NrTP-6 analog, in which the near-infrared dye DY676 was coupled to an extra N-terminal cysteine residue in NrTP-6 (DY676-Cys-NrTP6, “mini-crotamine”) efficiently penetrated and accumulated in certain lines and cancer cell populations, rendering this NrTP version a promising theranostic probe for imaging cancer heterogeneity and delivery of chemotherapeutics into subpopulations of cancer cells [152].

#### 2.5.2. Crotalicidin and Elapid CRAMPs

Crotalicidin (KRFKKFFKKVKKSVKKRLKKIFKKPMVIGVTIPF) is a 34-residue, linear, lysine-rich, amphipathic α-helical peptide from the venom gland of the South American rattlesnake C. d. terrificus, which shares high similarity with homologous sequences from the venom gland of diverse species of South American pit viper snakes, such as batroxicidin, lachesicidin and lutzicidin, with only a small number of conserved amino acid substitutions; these are collectively named vipericidins [153]. These peptides belong to a family of antimicrobial peptide-denominated CRAMPs (cathelicidin-related antimicrobial peptides), in which members are mature carboxy-terminal products, resulting from the proteolytic cleavage of a longer cathelicidin precursor that includes a signal peptide and a cathelin preproprepeptide, like the human cationic 18 kDa AMP (hCAP18/LL-37) [154]. Vipericidins, in general and crotalicidin, in particular, are surprisingly similar to previously characterized Asian elapid CRAMPs, despite South American pit vipers and Asian elapids (cobras and kraits) being geographically and phylogenetically distant venomous snake groups, which evolutionarily diverged millions of years ago. Multiple biological activities have been reported for crotalicidin, vipericidin-related sequences and elapid CRAMPs, with emphasis on antipathogenic and antitumoral properties, as reviewed elsewhere [135,155,156,157]. The antimicrobial activity ranges from multidrug-resistant clinical isolates to life-threatening viruses, as reported for cathelicidin-OH30 derived from the venom of the king cobra Ophiophagus hannah, as well as venom cathelicidin-BF30 and short derivatives from the banded krait Bungarus fasciatus venom [158,159]. The anti-inflammatory properties of venom cathelicidins have also been observed in in vitro and in vivo models of inflammation for cathelicidin-BF30 [160] and for CRAMPs from the venom of the blue-banded sea snake Hydrophis cyanocyntus, demonstrating the potent antimicrobial activity, associated with immunomodulatory effects of snake venom cathelicidins [161,162]. Short peptide fragments of snake venom cathelicidins have been prepared, displaying different levels of activity and efficacy against a range of microbes, including viruses [163], multidrug-resistant bacteria, pathogenic yeasts and biofilms [164,165,166,167].

The interaction of crotalicidin and elapid CRAMPs with biological membranes and biomimetics has been demonstrated for native, full sequences and short-derived peptide fragments and the ability for plasma membrane perturbation is associated with the mechanism of antimicrobial activity [168,169,170,171,172], as well as their antitumor effects, as determined experimentally in vitro with a variety of cancer cell lines [173,174]. Indeed, the cell penetration ability after membrane permeation has been verified for some venom cathelicidins and downsized analogs. For instance, Cbf-K16 and Cbf-A7A13, that is, cathelicidin- BF30, in which Asp at position 16 was substituted for K and Lys7 and Arg13 were replaced with Ala residues, was shown to bind with high affinity to bacterial DNA after penetrating the cytoplasmic membrane in vitro [175]. The fluorescent-labeled crotalicidin and the parental dodecapeptide (Ctn[15,16,17,18,19,20,21,22,23,24,25,26,27,28,29,30,31,32,33,34], KKRLKKIFKKPMVIGVTIPF-amide), conjugated with rhodamine B and/or fluorescein, accumulated in the plasma membrane, cytoplasm and the nucleus of prokaryotic and eukaryotic cells (pathogenic yeasts and tumor cells) [170,176]. Moreover, the internalization and interaction with intracellular targets, metabolic pathways and signaling reportedly contributed to the underlying mechanisms of action of native crotalicidin and short peptide derivatives [174,176]. These multiple effects of membrane permeation, cellular internalization and interaction with subcellular targets, pathways and signaling reportedly reinforce the selective microbial killing and selective cytotoxicity against cancer cells over healthy cells. Therefore, snake venom cathelicidins and analogs are an interesting class of peptides with cell penetration properties for development and application in biomedicine and pharmaceutical biotechnology.

#### 2.5.3. Cardiotoxin

Cardiotoxins comprise a group of membrane-interacting, three-finger cationic toxins, with approximately 60-residue, found in the venom of different species of cobra (e.g., Naja atra, N. oxiana and N. mossambica mossambica), which are cytotoxic to several eukaryotic cells. A mechanistic feature of interaction distinguishing cardiotoxins from other membrane-active α-helical amphipathic peptides and toxins is the fact that the hydrophobic tips of their β-sheet loops in the three-finger structures are inserted into membranes [177]. Accumulating experimental evidence has indicated that the biological effects of cardiotoxins are not completely dependent on direct plasma membrane damage but also involve modulation of intracellular signaling and, notably, cell penetration and disruption of organelle structures and functions, such as lysosomes and mitochondria [178,179,180]. For instance, cardiotoxins from the venoms of monocled cobra N. kaouthia and Caspian cobra N. oxiana, conjugated to rhodamine, were localized mainly in the lysosomes of human lung adenocarcinoma (A549) and promyelocytic leukemia (HL60) cells after membrane translocation and organelle accumulation [179], whereas rhodamine-labeled cardiotoxin VII4, derived from the Mozambique spitting cobra N. mossambica mossambica, translocated to the mitochondria and induced mitochondrial fragmentation in mouse primary cortical neurons and human neuroblastoma (SH-SY5Y) cells [180].

#### 2.5.4. Phospholipase A_2_

Secreted phospholipases A_2_ (sPLA_2_) are venom components widely distributed in organisms of all kingdoms, from cnidarians and insects (bees, wasps and ants) to arachnids and venomous snakes [181,182,183,184]. Based on their structure-activity, venom PLA2 are classified into distinct types that include enzymes that cause hydrolysis of glycerophospholipids and damage cell membranes, as well as toxins devoid of enzymatic activity that cause neurotoxicity and immunomodulation, inflammation and pain. Mendes and collaborators prepared short cationic (13-mer) peptides derived from the C-terminal region of the Lys49 PLA2 from the venom of the broad-banded copperhead snake Agkistrodon contortrix laticinctus, by replacing the natural lysine residues (KKYKAYFKFKCKK-amide) with arginine (RRYRAYFRFRCRR-amide); this imparted this engineered peptide characteristics of CPPs and AMPs and revealed an effective leishmanicidal activity [185].

#### 2.5.5. BPP-like

Bradykinin-potentiating peptides (BPPs) comprise a family of 5- to 14-residue proline-rich peptides found in the venom of pit vipers, wasps and frog skin. The main biological activity of these oligopeptides is the inhibition of the angiotensin-converting enzyme of the angiotensin system that regulates human blood pressure and, consequently, induces the severe hypotensive effect of the *Bothrops* venom [186]. Using a combination of size exclusion chromatography and liposome uptake, Sciani and colleagues [187] identified BPP-13a (<EGGWPRPGEIPP, where <E is pyroglutamic acid) from the *B. jararaca* venom that demonstrated potential cell-penetrating abilities. To confirm this finding, a fluorescent analog was prepared by conjugation of AlexaFluor^®^488 with desPyroglutamic1-BPP13a (desPyr-BPP13a; H2N-GGWPRPGPEIPPOH) analog and translocation into the cytoplasm of human melanoma tumor (SK-MEL-28 and A2058) cells was observed by fluorescence microscopy, with no detectable cytotoxicity.

Table 4 summarizes examples of peptides and derivatives from fish and amphibian secretions and snake venom, with cell-penetrating properties and cargo delivery capacity.

## 3. Discussion

In nature, a wide variety of toxins from microbial and animal origins affect eukaryotic cells either by interfering directly with and disrupting the cytoplasmic membrane or through interaction with integral membrane components, such as receptors and membrane microdomains, to gain access to the cytoplasm and interfere with subcellular structures and intracellular signaling. Well-known examples include the bacterial pore forming toxins (PFTs) and binary bacterial toxins. In the first group, single chain toxins with amphipathic α-helices (α-PFTs) or amphipathic β-hairpins (β-PFTs) oligomerize on phospholipid membranes and form pores or channels through which cellular contents extravasate. These cytolytic bacterial toxins are produced and secreted by a large number of Gram-negative and Gram-positive bacteria [188]. In the second group, in-solution formed after activation or preformed “A-B” binary toxins act by binding to their correspondent receptors through the “B” docking domain and translocate a catalytic “A”’ component into the cytosol, which disrupts ribosome structure and function and consequently protein synthesis of targeted cells [189]. Pore-forming cytolysins are also found in a variety of animal venoms, particularly in venom from arachnids (scorpion and spiders), hymenopterans (ants, bees and wasps) [190,191,192] and cnidarians (anemones, corals and jellyfishes), including zoanthid toxins belonging to the superfamily of membrane attack complex-perforin/cholesterol-dependent cytolysins (MACPF/CDC) [193,194,195]. Concurrently, antimicrobial peptides from diverse biological tissues, secretions and organisms of all kingdom also exert their effect essentially by membrane permeation and therefore, share a convergent mechanism of action with PFTs, despite their distinct target cell selectivity [196]. Thus, microbial PFTs, membrane-disruptive (cytolytic) venom peptides and AMPs that accumulate initially on the membrane surface by electrostatic attraction and/or hydrophobic interactions, form pores, permeabilize membranes and eventually enter into target cells and act intracellularly, in some cases [40,197]. In contrast, a handful of binary bacterial toxins and some plant toxins deliver the intracellular active toxin domain into the cytoplasm of target cells via receptor-mediated endocytosis and various intracellular trafficking pathways [189,197,198,199].

Non-disruptive membrane translocation and cell penetration of venom peptides are less common, with some examples emerging in the last decades. Several of these venom peptides that can penetrate cells by direct translocation and/or receptor-dependent and/or receptor-independent endocytosis to exert their effect on intracellular targets were presented herein. In addition, venom peptides that can penetrate cells by membrane-disruptive means, such as anoplin, lycosin-1 and melittin from arthropod venom; pardaxin from fish secretion; and crotalicidin, from snake venom, were also listed. Importantly, native animal venom peptides and toxins with cell-penetrating activity have been characterized and CPPs derived from them conceived through a variety of structural modifications of their native sequences. These structural modifications of cell-penetrating venom peptides include peptide downsizing, amino acid residue replacement, structural stabilization and production of chimeras, which mostly reduced peptide cytotoxicity, maintained the target selectivity and enhanced the cellular penetrability and the capacity of intracellular cargo transport and delivery. Undoubtedly, a distinction should be made between pore-forming, cytolytic peptides that can gain eventual entry into the cells and toxins through disruptive mechanisms from venom peptides and analogs that penetrate cells by non-disruptive, direct translocations and receptor-mediated and receptor-independent endocytosis. Despite this, both classes of membrane-interacting toxins can be engineered to excise the peptide motif responsible for membrane interaction and translocation, as observed in several examples presented herein.

## 4. Conclusions

Animal venoms are a blend of bioactive peptides, among other organic and enzymatic components, that in combination and synergistically disrupt the physiological processes of prey and/or victims. Apart from several families of bioactive peptides and proteins that act in a variety of biological processes and systems, such as neurotransmission, blood coagulation, immune modulation and tissue integrity, venom peptides and toxins with the intrinsic ability to penetrate cells by membrane-disruptive and non-disruptive mechanisms have been disclosed. These cell-penetrating venom peptides and toxin derivatives have expanded the library of animal toxins that act intracellularly and have presently served and will be useful in future, for biomedical and biotechnological applications.

## 5. Future Direction

The designed CPP from venom peptides have found applications in biomedicine and biopharmaceutical biotechnology, in diagnostics to detect populations of diseased cells or tissues, in therapy to induce selective cell death through molecular deliver of cell-impermeant drugs, organic compounds, radionuclides and crystal and metallic nanoparticles. By combining information on the cell penetrability of venom peptides and derivatives, it is expected this review will draw more attention to animal toxins and venom peptides that act intracellularly and promote their own passage across biological membranes to exert their effects. Therefore, discoveries of cell penetrating venom peptides and animal toxins will continue to contribute to the basic and applied research in this expanding field of CPPs derived from components of poisonous and venomous animals.

## 6. Materials and Methods

The preparation of this systematic review followed the recommendation of the PRISMA Statement for Reporting Systematic Reviews and Meta-Analyses of Studies that Evaluate Health Care Interventions: Explanation and Elaboration [200]. The search terms were: “cell penetrating peptide AND animal toxin” that yielded 140 entries from PUBMED. After selecting most relevant reports that adhered to the scope of this review, 50 general articles were excluded, including studies about “crotamine.” Considering the terms “crotamine AND cell-penetrating peptides” as search items, 26 additional articles were included. Then, the database was again queried to find fundamental and general studies on representative venom peptides with cell-penetrating properties that were found in the initial search, to obtain more complete information about their respective animal venom sources, structural features and main pharmacological activities, resulting in the final references, as listed in this manuscript.

## Figures and Tables

**Table 1:** Examples of cell-penetrating peptide (CPP) archetypes, origins, sequences, secondary structures and physicochemical classes.

CPP	Origin	Sequence	Physicochemical Class and Secondary Structure
Protein-derived			
TAT^48-60^	Protein of HIV-1	GRKKRRQRRRPQ	cationic random coil (rdc) *
Penetratin (Antp^43-68^)	Antennapedia homeodomain of *D. melanogaster*	RQIKIWFQNRRMKWKK	cationic β-strand/rdc *
VP22	Herpes simplex virus type I	NAKTRRHERRRKLAIER	amphipathic α-helix
pVEC	Cadherin^615–632^	IRKQAHAHSK	amphipathic β-strand/rdc *
Chimeric			
Transportan	Galanine/Mastoparan	GWTLNSAGYLLGKINLKALAALAKKIL	amphipathic α-helix *
Pep-1	HIV-reverse transcriptase/SV40 T-antigen	KETWWETWWTEWSQPKKKRKV	amphipathic α-helix *
MPG	HIV-gp41/SV40 T-antigen	GALFLGFLGAAGSTMGAWSQPKKKRKV	amphipathic β-strand/rdc
Synthetic			
Polyarginines	Based on Tat peptide	Poly-arginine, (R)_n_; 6 < *n* < 12	cationic rdc *
MAP	designed	KLALKLALKALKAALKLA	amphipathic α-helix *
KALA	designed	WEAKLAKALAKALAKHLAKALAKALKACEA	amphipathic α-helix

Note: Examples are based on references [17,19,20]. The mechanisms of membrane translocation and cell entry of these archetypal CPPs are not definitively elucidated. However, distinct mechanisms are involved, such as direct translocation (non-endocytic pathway, like an inverted micelle, pore formation, carpet-like and membrane thinning) and endocytosis-mediated internalization (macropinocytosis, clathrin- and caveolin-dependent endocytosis, clathrin- and caveolin-independent endocytosis and receptor-mediated endocytosis. These CPPs can utilize more than a single route for cell entry [19,20,30]. “*”, as structurally characterized in the presence of negatively charged dioleoylphosphatidylglycerol [26].

**Table 2:** Examples of peptides from insect (honey bee and wasp) venom and derivatives with cell penetrating property.

Peptide	Sequence ^a^	Size	Major Structural Features ^b^	Mechanism(s) of Cell Penetration ^c^	Cargo Delivery ^d^	Ref.
Honey bee						
Mellitin (MEL)	GIGAVLKVLTTGLPALISWIKRKRQQ-NH2	26	Amphipathic α-helix	DT, PF	FD	[49]
Tat/CM18 hybrid			Chimera	DT, MD, ND	CI	[52]
MEL-d(KLAKLAK)_2_	GIGAVLKVLTTGLPALISWIKRKRQQGGGGS-d[KLAKLAKKLAKLAK]	45	Chimera	DT (?)	AP	[53]
p5RHH	VLTTGLPALISWIRRRHRRHC	21	C-terminal fragment	Endocytosis (?)	DNA polyplexes	[54,55]
p5RWR	VLTTGLPALISWIKRKRQQRWRRRR	25	C-terminal fragment	Endocytosis (?)	DNA polyplexes	[54,55]
MT20	GIGAVLKVLTTGLPALISWI	20	Hydrophobic helix	DT, MD	DNA polyplexes	[56]
FL20	GIGAILKVLATGLPTLISWI	20	Hydrophobic helix	DT, MD	DNA polyplexes	[56]
Melittin [1,2,3,4,5,6,7,8,9,10,11,12,13,14]	GIGAVLKVLTTGLP	14	N-terminal fragment	DT	NC, LP	[57]
						
Wasp						
Anoplin	GLLKRIKTLL-NH_2_	10	Amphipathic α-helix	DT, PF	FD	[60]
Anoplin dimer	(GLLKRIKTLL-NH_2_)_2_	20	C-N terminal dimer	DT, PF	-	[61]
Mastoparan	INLKALAALAKKIL-NH_2_	14	Amphipathic α-helix	DT, PF	-	[64]
Mastoparan-X	INWKGIAAMAKKLL-NH_2_	14	Amphipathic α-helix	DT, PF	-	[65]
Transportan	GWTLNSAGYLLGKINLKALAALAKKIL-NH_2_	27	Chimera	multiple	CI	[66]
TP10	AGYLLGKINLKALAALAKKIL-NH_2_	21	Shorter transportan	multiple	CI	[70,77]
TP10-5	AGYLLGKINLKKLAKL(Aib)KKIL-NH_2_	21	Helical stabilized	DT	FD	[71]
Transportan 9dR	GWTLNSAGYLLGKINLKALAALAKKIL(dR)9	36	Chimera	Endocytosis (?)	siRNA	[73]
Mitoparan	INLKKLAKL(Aib)KKIL-NH_2_	14	Mastoparan analogue	DT	FD, AP	[78,79,80]

Notes: ^a^-NH2, amidated peptide; anoplin C-N terminal dimer, as prepared by intermolecular triazole bridge; Aib, α-aminoisobutyric acid; ^b^ Particular structural characteristics of native, designed or analogous peptides; C-N terminal dimer, intermolecular dimerization involving the carboxyl-terminus of one peptide and the amino-terminus of the other; ^c^ DT, direct translocation; PF, pore forming; MD, membrane-disruptive cell uptake; NR, non-disruptive penetration; RM, receptor-mediated endocytosis; HI, hydrophobic insertion; multiple, more than a single mechanism of cell entry, that can involve direct translocation and endocytosis; ^d^ FD, fluorescent dyes; CI, diverse types of cell impermeant cargoes; AP, apoptotic peptides; NC, nanocrystals; LP, large proteins; RD, radionuclides; EZ, enzymes; IR, infrared; “-,” not applicable.

**Table 3:** Examples of peptides and derivatives from arachnid venoms with cell-penetrating and cargo delivery capacity.

Peptide	Sequence	Size	Major Structural Features	Mechanism(s) of Cell Penetration	Cargo Delivery	Ref.
Spider						
Latarcin-1 (Ltc1)	SMWSGMWRRKLKKLRNALKKKLKGE	25	cationic α-helix	DT, PF	FD	[81]
Ltc1-decapeptide (LDP)	KWRRKLKKLR	10	Designed	DT	FD	[82]
LDP-NLS	KWRRKLKKLRPKKKRKV	17	Chimera	ED	FD, EZ	[83]
Lycosin-I	RKGWFKAMKSIAKFIAKEKLKEHL	24	Amphipathic α-helix	DT, ND	FD, NP	[87,88]
R-lycosin-I	Ac-RGWFRAMRSIARFIARERLREHL-amide	24	Lys→Arg analogue	ED	FD	[89]
						
Scorpion						
Chlorotoxin (CTX)	MCMPCFTTDHQMARKCDDCCGGKGRGKCYGPQCLCR	36	ICKfold/knottin	ED	FD, RD	[91,92,93]
[K15R/K23R]CTX	MCMPCFTTDHQMARRCDDCCGGRGRGKCYGPQCLCR	36	Mutant analogue	ED	FD	[95]
[K15R/K23R/Y29W]CTX	MCMPCFTTDHQMARRCDDCCGGRGRGKCWGPQCLCR	36	Mutant analogue	ED	FD	[95]
Maurocalcine (MCa)	GDCLPHLKLCKENKDCCSKKCKRRGTNIEKRCR	33	ICK fold/knottin	DT, ND	CI, NP	[99,100,101]
MCaUF1–9-C	GDAbuLPHLKLC	10	Truncated, unfold	DT	FD	[105]
Imperatoxin A (IpTxa)	GDCLPHLKRCKADNDCCGKKCKRRGTNAEKRCR	33	ICK fold/knottin	n	FD	[109]
Hadrucalcin (HdCa)	SEKDCIKHLQRCRENKDCCSKKCSRRGTNPEKRCR	35	ICK fold/knottin	DT, ND	-	[110]
HadUF_1−11_ (H11)	SEKDAbuIKHLQR-C	12	N-terminal fragment	DT, ND, ED (?)	NP	[111]
WaTx	ASPQQAKYCYEQCNVNKVPFDQCYQMCSPLERS	33	CS-helical hairpin	DT, ND	FD	[112]

Notes: Ac, acetylated; -NH_2,_ amidated; Abu, Lys→Arg replacement; C-S, cysteine stabilized α-helix; DT, direct translocation; PF, pore forming; ED, endocytosis;; ND, non-disruptive penetration; RM, receptor-mediated endocytosis; HI, hydrophobic insertion; multiple, more than a single mechanism of cell entry, that can involve direct translocation and endocytosis; “n“, not informed; FD, fluorescent dyes; EZ, enzymes; NP, nanoparticles; RD, radionuclides; CI, diverse types of cell impermeant cargoes; “-“, not applicable.

**Table 4:** Examples of peptides and derivatives from fish and amphibian secretion and snake venom, with cell-penetrating and cargo delivery capacity.

Peptide	Sequence ^a^	Size	Major Structural Features ^b^	Mechanism(s) of Cell Penetration ^c^	Cargo Delivery ^d^	Ref.
Fish						
Ac-Pardaxin P5	Ac-GFFALIPKIISSPLFKTLLSAVGSALSSSGDQE	33	Hydrophobic helix	DT, PF	FD	[117]
						
Amphibian						
bombesin	EQKLGNQWAVGHLM-NH_2_	14	helical harpin	RM	-	[118,119,120]
Tat-K3-bombesin^1-14^	RKKRRQRRRGGCGEQKLGNQWAVGHLM-NH_2_	27	Chimera	RM	RD	[124]
						
Snake						
Crotamine	YKQCHKKGGHCFPKEKICLPPSSDFGKMDCRWRWKCCKKGSG	42	β-defensin fold	multiple	FD, CI	[138]
NrTP1	YKQCHKKGGKKGSG	14	N↔C splice variant	multiple	FD	[146,147]
NrTP6	YKQSHKKGGKKGSG	14	NrTP1 CysΔSer	multiple	FD, EZ	[148]
DY676-NrTP6	C-YKQSHKKGGKKGSG	15	Extra Cys		IR	[152]
CyLop-1	CRWRWKCCKK	10	Encrypted sequence	DT, ED	FD	[145]
						
Crotalicidin	KRFKKFFKKVKKSVKKRLKKIFKKPMVIGVTIPF	34	Amphipathic α-helix	DT, PF	FD	[176]
Ctn[15,16,17,18,19,20,21,22,23,24,25,26,27,28,29,30,31,32,33,34]	KKRLKKIFKKPMVIGVTIPF-NH_2_	20	C-terminal fragment	DT, PF	FD	[170,176]
cathelicidin- BF30	KFFRKLKKSVKKRAKEFFKKPRVIGVSIPF	30	Shorter analogue	DT, PF	-	
Cardiotoxin	LKCNKLIPLAYKTCPAGKNLCYKMFMVSNKTVPVKRGCID ACPKNSLLVKYVCCNTDRCN	60	Three-finger fold	DT, HI	FD	[179]
PLA_2_ C-terminal	KKYKAYFKFKCKK-NH_2_	13	C-terminal fragment	DT, MD	-	[185]
PLA_2_ fragment K→R	RRYRAYFRFRCRR-NH_2_	13	Mutant K→R	DT, MD	-	[185]
BPP-13a	<EGGWPRPGEIPP	12	Native	DT (?)	-	[187]
desPyr-BPP13a	GGWPRPGPEIPP	12	Mutant analogue	DT (?)	FD	[187]

Notes: ^a^ Ac, acetylated; -NH_2,_ amidated; <E, pyroglutamic; DT, direct translocation; ^b^ N↔C, spliced derivative of crotamine, comprising the N-terminal residues 1 to 9 and C-terminal 38 to 42 ([1,2,3,4,5,6,7,8,9] **~** [38,39,40,41,42]); NrTP6, a NrTP1 with a Cys-Ser replacement; DY676, near-infrared dye; ^c^ DT, direct translocation; PF, pore forming; RM, receptor-mediated endocytosis; multiple, more than a single mechanism of cell entry, that can involve direct translocation and endocytosis; ED, endocytosis; HI, hydrophobic insertion; MD, membrane-disruptive cell uptake; ^d^ FD, fluorescent dyes; RD, radionuclides; EZ, enzymes; IR, infrared dye; “-“ not applicable;.

## Data Availability

Data sharing not applicable.
